# Management of suicide and self-harm risk by the National Mental Health Helpline in the State of Qatar

**DOI:** 10.1192/bjo.2023.70

**Published:** 2023-05-25

**Authors:** Majid Alabdulla, Yousaf Iqbal, Hadeel Gafar Ali Mohamed, Dhanya Shinith, Rodel Austria Buenaventura, Katja Anneli Warwick Smith, Mohamed Hamideh, Sami Ouanes

**Affiliations:** Hamad Medical Corporation, Doha, Qatar; and College of Medicine, Qatar University, Doha, Qatar; Hamad Medical Corporation, Doha, Qatar

**Keywords:** Helpline, mental health, self-harm, suicide, COVID-19

## Abstract

**Background:**

Suicide is a serious public health problem.

**Aims:**

To investigate the sociodemographic and clinical features of callers (patients) classed by the Qatar National Mental Health Helpline (NMHH) as moderate to high priority based on the risk of self-harm or suicide during the COVID-19 pandemic.

**Method:**

The study design was a retrospective chart review of patients who contacted the helpline in the first 12 months, starting 1 April 2020. Data of those classed as moderate to high priority based on risk to self were collected using a specifically designed form. Absolute and relative frequencies for each of the studied categorical variables were determined.

**Results:**

Four hundred and ninety-eight patients were included. More than half were female. The mean age was 32 years (range 8–85 years). Two-thirds of patients were from Arab countries and more than half of all patients had contacted mental health services for the first time. The most common symptoms elicited included suicidal thoughts, depressed mood and disturbed sleep. The most common psychiatric disorders were depression and generalised anxiety disorder. Most patients were seen within 4 h and received psychiatric interventions. Virtually all patients received non-pharmacological interventions; only 38.5% received pharmacological interventions. The majority had follow-up appointments arranged with mental health services.

**Conclusions:**

People from the Indian subcontinent and males proportionally approached services less, which may reflect stigma. The NMHH improved access to care for patients considered at risk to self and prevented hospital admissions. The NMHH offers a valuable additional choice to patients and assists in prevention and management of suicidal behaviour and other mental health difficulties.

The COVID-19 pandemic is associated with increased mental health difficulties.^1^ A meta-analysis of studies published during its first year suggested increased rates of suicidal ideation and suicidal and self-harm attempts.^2^ Suicide is a huge public health concern. According to the World Health Organization, more than 700 000 people die by suicide every year. For every completed suicide, there are many more people who attempt suicide.^3^ In Qatar, the annual incidence of suicide attempts was found to be 159/100 000 patients visiting the emergency department and 14.2/100 000 in the general population.^4,5^ A quarter of all patients presenting with mental health problems to emergency departments displayed suicidal behaviour.^5^ Following a self-harm attempt, there is a significant risk of future suicide.^6^

Studies have outlined some sociodemographic and clinical features associated with a higher risk of suicide and self-harm. A systematic review identified suicide risk factors as any mental illness, prior suicidal behaviour, substance misuse, physical health problems, genetic predisposition and unemployment.^7^ Another study also found economic adversity due to the global recession to be a suicide risk factor.^8^ A more recent systematic review identified mental illness, especially depression, presentation with self-harming attempts, previous psychiatric hospital admissions, physical health problems and substance misuse as risk factors.^9^ In Qatar, single expatriate males were found to be at a higher risk of suicide and Arabs at a higher risk of attempting suicide.^4^ However, rates of suicidal behaviour were not higher among low-paid economic migrants.^5^ A further study identified men over 25 years of age, widowed or divorced and/or with psychotic disorders as more likely to engage in near-fatal self-harm.^10^

One of the strategies of suicide prevention includes early identification, assessment, management and follow-up of people with suicidal behaviour.^11^ Suicide prevention hotlines are effective as they offer immediate safety to callers and link about 44% of callers with appropriate mental health service resources. Studies show that callers had lower suicidality, hopelessness and psychological pain after contact with a hotline.^12–14^ A study in Taiwan also highlighted a significant reduction in callers’ emotional distress and suicidality during the course of the session and concluded that the hotlines are useful suicide prevention and crisis intervention services.^15^ Telemedicine and hotline services started to play a significant role during the COVID-19 pandemic.^16^

## The National Mental Health Helpline

Qatar implemented a holistic plan to manage the pandemic, including telepsychiatry and the National Mental Health Helpline (NMHH) service.^16^ The NMHH was launched in the State of Qatar in April 2020 to facilitate easy access to high-quality care during the COVID-19 pandemic. This was a result of collaborative efforts of the Hamad Medical Corporation Mental Health Service, the Ministry of Public Health and the Primary Health Care Corporation.^17,18^ It aimed to provide callers with a one-stop free telephone service 6 days a week by calling a dedicated number, 16000. It offered a multidisciplinary mental health service and was widely publicised. The NMHH team included trained and highly skilled mental health professionals consisting of psychiatrists, psychologists, psychiatric nurses and triage clinicians. Diverse and culturally competent team members could communicate in Arabic, English, Hindi, Urdu, Malayalam and Pashtu, among other languages. A total of 157 staff were trained to offer helpline care. The team offered easy access to a confidential service embedded in biopsychosocial interventions including assessments and brief interventions for children and adolescents, adults and older adults.

The NMHH employed a triage system where all the calls were handled by trained and qualified mental health professionals (nurses or psychologists). All members received standardised training on triage assessments and were deemed competent in the defined competency before taking calls autonomously. Triage coordinators offered supervision to maintain consistency and high quality at all times. The Patient Health Questionnaire-9 and General Anxiety Disorder-7 scales were used if deemed appropriate, otherwise triage primarily relied on clinical judgement based on history, mental state examination and risk assessment. Initial triage assessments were tailored to the nature of the callers’ problems, whereas subsequent comprehensive assessment and management were undertaken by professionals from the appropriate discipline. A situation, background, assessment and recommendation (SBAR) format, with a set of standardised prompt questions, was implemented to ensure consistency in the triage.

Calls were classified into mild, moderate or high priority to determine the urgency of response, based on the risk of harm to self or others, aggressive behaviour, the nature and severity of psychiatric symptoms, and protective factors. A triage risk assessment framework (Table 1), developed with guidance from the Victorian Mental Health Triage Scale,^19^ was used.

**Table 1:** Triage risk assessment framework

Level of priority	Description
High	Emergency: immediate intervention (e.g. overdose, in the middle of a suicide attempt, violent at the time/threats with possession of a weapon) Very urgent: within 2 h (e.g. suicidal ideation with a plan and means, with family support and feels safe to wait for a doctor to call back, or person actively hallucinating but not presenting an immediate risk to others and is under supervision)
Moderate	Within 8 h (e.g. suicidal ideation with no plan and/or history of suicidal ideation, rapidly increasing symptoms of psychosis, high-risk behaviour associated with perceptual disturbance, delirium, dementia and impaired impulse control)
Low	Within 72 h (e.g. carer distress associated with the person's mental illness, early signs of psychosis, low-grade mood or anxiety, medication refills or deliveries and general enquiries)

Over recent years, Qatar has seen an accelerated growth of in-patient, community, out-patient and specialised mental health services supported by the National Health Strategy.^20–22^ There are dedicated in-patient facilities for the management of psychiatric conditions and substance use disorders. Furthermore, there are residential rehabilitation services and dedicated liaison psychiatry services in major acute general hospitals. Community mental health teams and out-patient services for people of all ages have seen expansion to facilitate care outside the hospital. Additionally, specialist services include child and adolescent mental health, old age psychiatry, women's mental health, intellectual disability and dedicated migrant workers’ services.^23^ Primary care mental health services are delivered by general practitioners. The NMHH service caters for people of all ages experiencing diverse mental conditions. There is a need to develop a dedicated crisis resolution and home-based treatment team.

One of the aims of the NMHH was to offer prompt assessment and intervention to people who called the service in crisis and carried risks of harm to themselves. This initiative formed part of wider efforts of suicide prevention by the State of Qatar.^20^

A patient satisfaction survey of NMHH demonstrated predominantly positive feedback and 90% of callers stated that they would recommend the service to a friend or a relative.^24^ NMHH was awarded a Service Delivery Award by the World Health Organization's Eastern Mediterranean Regional Office in recognition of its concerted efforts in addressing mental health and well-being issues for the Qatari community during the COVID-19 pandemic.^25^

## Study aims

This study aimed to investigate the sociodemographic and clinical profiles of a sample of NMHH callers (patients) who were classed as moderate to high priority based on the risk of self-harm or suicide by the NMHH during the COVID-19 pandemic. It also aimed to describe factors associated with high-risk prioritisation and interventions offered to these individuals.

## Method

The study design was a retrospective case-note review. The study was conducted across the National Mental Health Helpline (NMHH) services in the State of Qatar among patients who contacted the helpline in its first 12 months, starting 1 April 2020.

The inclusion criteria were: patient of any age who was assessed by the NMHH and classed as moderate to high priority due to the risk of self-harm or suicide.

Exclusion criteria included: patients whose cases were classed as a low priority.

A search of the secure electronic database held by NMHH services was conducted to identify all cases highlighted as a moderate or high priority. Further screening of cases was done using keywords, including suicide, suicidal, self-harm, overdose, death wishes and hopelessness, to identify patients at risk of harm to self. All eligible cases during the study period were included.

Data were collected from the database using a specifically designed form by three researchers and regular meetings were held to ensure consistency in the data collection. Any uncertainty was resolved by discussion with a fourth team member, who was overseeing the data collection.

The data collection form included information on demographic characteristics, whether the patient was known to mental health services and history of mental health problems, suicidal thoughts, self-harm, suicide attempt or aggressive behaviour. It also included information on substance misuse, current psychiatric symptoms, life stressors and current occupational status. Lastly, information was collected about the contact with NMHH: pharmacological and non-pharmacological interventions (psychoeducation and brief psychological intervention), NMHH professional groups that offered the interventions, waiting time before seeing/speaking with the professionals and time it took to conclude the initial management.

### Statistical analysis

Data analysis was conducted using SPSS version 26 for Windows. We determined absolute and relative frequencies (percentages) for each of the studied categorical variables.

We also conducted binary logistic regressions:

- using priority (high versus moderate) as a dependent variable and with the following independent variables: age, gender, citizenship (Qatari versus non-Qatari), being known to mental health services, substance misuse, psychiatric history, history of suicide attempt, depression, generalised anxiety disorder, psychotic disorder and personality disorder;
- using intervention by a psychiatrist (yes versus no) or a psychologist (yes or no) as a dependent variable, and with the following independent variables: age, gender, citizenship (Qatari versus non-Qatari), being known to mental health services, substance misuse, psychiatric history, history of suicide attempt, depression, generalised anxiety disorder, psychotic disorder, personality disorder and social stressors (as a dichotomous variable).

*P*-values were adjusted for multiple comparisons using Bonferroni's method. The alpha value was set at 0.05 for all analyses.

### Ethics approval and patient consent

The study was approved by the Hamad Medical Corporation Institutional Review Board (MRC-01-21-322). All procedures contributing to this work comply with the ethical standards of the relevant national and institutional committees on human experimentation and with the Helsinki Declaration of 1975, as revised in 2008. Individual patient consent was waived by the IRB as the study design model was a retrospective review of case-notes anonymised before analysis.

## Results

During the 12-month study period, there were a total of 12 594 calls. Older adults and children and adolescents made up 6.4% and 2.7% of all calls respectively. Out of all the calls, 2.7% were classed as high and 13.6% as moderate priority calls.

In total, 498 calls fulfilled the study's inclusion criteria. The sociodemographic and clinical characteristics of included patients are detailed in Table 2. Out of included calls, roughly one-third were considered as high priority and two-thirds as moderate priority calls. There were slightly more females (57.3%, *n* = 285/497) than males (42.7%, *n* = 212/497).

**Table 2:** Sociodemographic and clinical features of participants (*n* = 498)

		*n*	%
Age range, years^a^	<18	38	7.7
	18–29	216	43.7
	30–39	112	22.7
	40–49	71	14.4
	50–59	46	9.3
	60–69	5	1.0
	70–79	4	0.8
	80–89	2	0.4
Gender^a^	Male	212	42.7
	Female	285	57.3
Ethnicity^a^	Non-Qatari Arab	217	43.9
	Qatari	125	25.3
	Indian	57	11.5
	Filipino	24	4.9
	Pakistani	12	2.4
	African	11	2.2
	Bengali	7	1.4
	Nepali	7	1.4
	Sri Lankan	5	1.0
	Others	29	5.9
Language	English	171	34.3
	Hindi	7	1.4
	Arabic	313	62.9
	Tamil/Malayalam	7	1.4
Caller	Patient	387	77.7
	Carer	100	20.1
	Other professional	11	2.2
Priority	Moderate	334	67.1
	High	164	32.9
Patient known to mental health services		218	43.8
History of self-harm^a^		128	36.1
History of suicide attempt^a^		102	28.7
History of suicidal thoughts^a^		198	51.3
History of aggressive behaviour^a^		36	12.2
Psychiatric history^a^		319	65.4
Pre-existing psychotropic medication^a^		224	49.2
Recent history of substance use^a^		27	8.0
Occupation^a^	Unemployed	44	23.7
	Student	35	18.8
	Housewife	14	7.5
	Employed	93	50.0

a. There are some missing data for these variables and, so, percentages are based only on the available data.

Median and mean ages were 29 and 32 years and the range was 8–85 years. Around two-thirds (66.4%, *n* = 328/494) were between 18 and 39 years of age. Most (69.2%, *n* = 342/494) were from Arab countries, and 17.8% (*n* = 88/494) were from the Indian Subcontinent. The most common primary languages spoken were Arabic (62.9%, *n* = 313) and English (34.3%, *n* = 171). The majority of calls were made by patients (77.7%, *n* = 387), followed by carers (20.1%, *n* = 100).

Less than half (43.8%, *n* = 218) of the patients were known to the Mental Health Services. 51.3% (*n* = 198/386) had a history of suicidal thoughts, and 28.7% (*n* = 102/355) had a history of suicide attempts.

The most common symptoms elicited included suicidal thoughts (73.1%, *n* = 364), depressed mood (65.3%, *n* = 325), disturbed sleep (58.6%, *n* = 292), anxiety (43.4%, *n* = 216), disturbed appetite (34.3%, *n* = 171) and death wishes (26.9%, *n* = 134) (Table 3).

**Table 3:** Most common symptoms reported by participants (*n* = 498)

Symptoms	*n*	%
Suicidal thoughts	364	73.1
Depressed mood	325	65.3
Disturbed sleep	292	58.6
Anxiety	216	43.4
Disturbed appetite	171	34.3
Death wishes	134	26.9
Loss of interest	102	20.5
Social isolation	98	19.7
Self-harm	85	17.1
Tiredness	74	14.9
Panic	69	13.9
Irritability	66	13.3
Tearfulness	59	11.8
Distress	53	10.6
Hopelessness/helplessness	53	10.6
Excessive worries	52	10.4
Emotional instability	50	10.0
Impaired concentration	49	9.8
Fear	44	8.8
Somatic symptoms	43	8.6
Aggression	41	8.2
Obsessions	29	5.8
Self-harm or suicide attempt	28	5.6
Hallucination	27	5.4
Weight loss	27	5.4
Thoughts of harm to others	26	5.2
Low confidence	24	4.8
Negative thinking	23	4.6
Relationship difficulties	23	4.6
Guilt	20	4.0
Paranoid thoughts	20	4.0
Palpitations	19	3.8
Abnormal/disinhibited behaviour	18	3.6
Elated mood	14	2.8
Compulsion	13	2.6
Suicide plan	12	2.4
Impulsiveness	11	2.2
Agitation	10	2.0
Delusion	9	1.8
Bodily aches and pains	8	1.6
Restlessness	8	1.6
Impaired memory	7	1.4
Dissociative symptoms	6	1.2
Chronic feelings of emptiness	6	1.2
Sexual problems	5	1.0
Pressured speech	3	0.6
Rumination	3	0.6

The most common psychiatric disorders were depression (30.5%, *n* = 152), generalised anxiety disorder (11.8%, *n* = 59), mixed anxiety and depression (11.6%, *n* = 58) and personality disorders (9.0%, *n* = 45) (Table 4).

**Table 4:** Prevalence of the most common psychiatric disorders among participants (*n* = 498)

	*n*	%
No formal diagnosis made	191	38.4
Depression	152	30.5
Generalised anxiety disorder	59	11.8
Mixed anxiety and depression	58	11.6
Personality disorder	45	9.0
Obsessive–compulsive disorder	26	5.2
Panic disorder	26	5.2
Substance use disorder	21	4.2
Adjustment disorder or acute stress reaction	16	3.2
Bipolar affective disorder	15	3.0
Other	11	2.2
Psychotic disorders	10	2.0
Neurodevelopmental disorders	7	1.4
Dementia or delirium	4	0.8
Post-traumatic stress disorder	4	0.8
Eating disorder	2	0.4
Somatisation disorder	2	0.4

The most common stressors included: relationship/family stress (49.9%, *n* = 173/347), limited social support (33.8%, *n* = 135/400), pandemic-related stress (29.3%, *n* = 91/311) and work-related stress (22.2%, *n* = 73/329) (Table 5).

**Table 5:** Most common stressors reported by participants

Life stressors	*n*	%^a^
Relationship/family stress	173	49.9
Limited social support	135	33.8
Pandemic-related stress	91	29.3
Work-related stress	73	22.2
Living away from family	69	16.5
Financial stress	21	7.0

a. There are some missing data and, so, percentages are based only on the available data. Data for each stressor is collected separately.

Most patients (82.1%, *n* = 408) received an intervention from a nurse. Almost three-quarters (70.9%, *n* = 353) had an intervention by a psychiatrist. One-third (34.9%, *n* = 174) had an intervention by a psychologist.

Virtually all patients (98.0%, *n* = 479) received non-pharmacological interventions; 38.5% (*n* = 188) received pharmacological intervention which included prescribing and/or discussing and advising psychotropic medication. The majority (83.2%, *n* = 412) were offered a follow-up appointment with mental health services. In-patient psychiatric admission was advised for 11.9% (*n* = 58) of patients.

Most (83.1%, *n* = 414) spoke to a professional within 4 h. Time under helpline care was <12 h in 79.8% of cases (*n* = 392) (Table 6).

**Table 6:** Interventions provided to participants (*n* = 498)

		*n*	%
Intervention done by psychiatrist		353	70.9
Intervention done by psychologist		174	34.9
Intervention done by nurse		408	82.1
Psychotropic medication intervention		188	38.5
Non-pharmacological strategies provided		479	98.0
Follow up in mental health hospital arranged		412	83.2
In-patient admission advised		58	11.9
Waiting time to speak with professional	<1 h	148	31.6
	1–4 h	266	56.7
	5–8 h	27	5.8
	>8 h	28	6.0
Time under helpline care	<12 h	392	79.8
	13–24 h	27	5.5
	1–3 days	66	13.4
	4–7 days	4	0.8
	>7 days	2	0.4

When comparing Qatari and non-Qatari participants, Qatari participants had a significantly higher male gender ratio and were more likely to be known to mental health services, to have a history of suicide attempts and to have been diagnosed with a psychotic disorder or a substance use disorder (Table 7).

**Table 7:** Comparisons between Qatari and non-Qatari participants^a^

		Non-Qatari		Qatari		
		*n*	%	*n*	%	*P*
Age, mean (s.d.)		32.4 (12.7)		30.6 (12.6)		0.178
Gender	Male	149	40.3	63	50.4	0.048
	Female	221	59.7	62	49.6	
Priority	Moderate	257	69.3	77	61.6	0.123
	High	114	30.7	48	38.4	
Patient known to mental health services		144	38.9	73	58.4	0.000
History of suicide attempt		71	25.9	31	38.8	0.026
Psychiatric history		228	63.0	89	71.8	0.081
Depression		116	31.3	36	28.8	0.654
Generalised anxiety disorder		39	10.5	19	15.2	0.197
Psychotic disorders		1	0.3	9	7.2	0.000
Personality disorder		30	8.1	14	11.2	0.281
Substance misuse		10	2.7	11	8.8	0.003
Social stressors		305	82.2	100	80.0	0.594
Intervention done by psychiatrist		257	69.6	94	76.4	0.204
Intervention done by psychologist		134	36.2	39	31.2	0.330
Intervention done by nurse		306	82.7	100	80.0	0.502
Psychotropic medication intervention received		140	38.4	46	38.0	1.000

a. There are some missing data so comparison analysis was done only on the available data.

Binary logistic regression showed that only history of suicide attempts and the presence of a psychotic disorder were significantly associated with a higher priority rating (*P* = 0.001; OR = 2.533 (95% 1.480–4.334) and *P* = 0.025, OR = 2.588 (95% CI 1.376–128.654) respectively) (Table 8).

**Table 8:** Binary logistic regression: factors associated with high priority

	*B*	s.e.	*P*	OR	95% CI for OR	
					Lower	Upper
Age	−0.003	0.011	0.779	0.997	0.977	1.018
Gender	−0.291	0.257	0.257	0.748	0.452	1.237
Qatari	0.100	0.301	0.740	1.105	0.612	1.994
Patient known to mental health services	0.190	0.322	0.554	1.210	0.644	2.272
Substance misuse	0.440	0.596	0.460	1.553	0.483	4.997
Psychiatric history	0.032	0.330	0.923	1.032	0.541	1.971
History of suicide attempt	0.929	0.274	0.001	2.533	1.480	4.334
Depression	0.193	0.266	0.467	1.213	0.721	2.043
Generalised anxiety disorder	−0.741	0.448	0.098	0.477	0.198	1.147
Psychotic disorder	2.588	1.158	0.025	13.307	1.376	128.654
Personality disorder	0.161	0.391	0.681	1.174	0.546	2.525

The presence of depression and the absence of social stressors were significantly associated with an intervention by a psychiatrist, whereas the presence of social stressors and the absence of a depressive disorder were associated with an intervention by a psychologist (Tables 9 and 10).

**Table 9:** Binary logistic regression: factors associated with an intervention by a psychiatrist

	*B*	s.e.	Wald	d.f.	Significance	Exp(*B*)	95% CI for exp(*B*)	
							Lower	Upper
Age	0.014	0.011	1.517	1	0.218	1.014	0.992	1.036
Gender	0.040	0.270	0.022	1	0.882	1.041	0.613	1.766
Qatari	−0.129	0.337	0.146	1	0.702	0.879	0.454	1.702
Patient known to mental health services	0.037	0.362	0.010	1	0.919	1.038	0.510	2.111
Substance misuse	20.204	9848.679	0.000	1	0.998	594 921 350.8	0.000	
Psychiatric history	0.458	0.357	1.645	1	0.200	1.581	0.785	3.181
History of suicide attempt	−0.331	0.306	1.175	1	0.278	0.718	0.395	1.307
Depression	1.183	0.314	14.236	1	0.000	3.265	1.766	6.038
Generalised anxiety disorder	0.777	0.491	2.507	1	0.113	2.174	0.831	5.687
Psychotic disorder	20.561	15249.743	0.000	1	0.999	850 251 059.5	0.000	
Personality disorder	0.133	0.471	0.080	1	0.778	1.142	0.454	2.873
Social stressors	−0.886	0.380	5.424	1	0.020	0.412	0.196	0.869

**Table 10:** Binary logistic regression: factors associated with an intervention by a psychologist

	*B*	s.e.	Wald	d.f.	Significance	Exp(*B*)	95% CI for exp(*B*)	
							Lower	Upper
Age	−0.012	0.010	1.334	1	0.248	0.988	0.969	1.008
Gender	−0.129	0.240	0.287	1	0.592	0.879	0.549	1.408
Qatari	0.003	0.292	0.000	1	0.992	1.003	0.566	1.777
Known patient to Mental Health	0.198	0.312	0.403	1	0.526	1.219	0.661	2.246
Substance misuse	−0.352	0.646	0.297	1	0.586	0.703	0.198	2.494
Past psychiatric history	−0.484	0.310	2.440	1	0.118	0.616	0.335	1.131
Past history of suicidal attempt	−0.337	0.278	1.473	1	0.225	0.714	0.414	1.230
Depression	−0.526	0.256	4.223	1	0.040	0.591	0.358	0.976
Generalised anxiety disorder	−0.130	0.368	0.124	1	0.724	0.878	0.427	1.807
Psychotic disorder	−20.747	15956.359	0.000	1	0.999	0.000	0.000	
Personality disorder	0.091	0.400	0.051	1	0.821	1.095	0.500	2.397
Social stressors	0.733	0.314	5.451	1	0.020	2.081	1.125	3.849

## Discussion

To our knowledge, this is the first major study to comprehensively describe the demographic and clinical characteristics of patients presenting with self-harm and suicide risks to the National Mental Health Helpline in the Middle East and North African region.

### General patient demograhics

Our study population's median and mean ages were 29 and 32 years respectively, which is consistent with the demographics of Qatar, which has a relatively young population with a median age of 32 years.^26^ About 8% of all patients were children and adolescents. NMHH mostly received initial calls from their carers and calls were managed by the dedicated child and adolescent mental health service (CAMHS) psychologists. The majority of these patients were later referred to the CAMHS for ongoing management.

The sample included patients from diverse ethnic backgrounds. The largest group of patients were non-Qatari Arabs. Qatari nationals represented over a quarter of all the patients, whereas they make up 11.6% of the total population.^27^ Proportionally more non-Qatari Arabs and Qatari citizens approached NMHH, which may reflect relatively increased risk, lower stigma or increased popularity of the NMHH among this group. However, a study from Dubai highlighted that the suicide rate was seven times higher among expatriates than nationals and over three-quarters of expatriates who died by suicide were Indian.^28^ Other factors associated with increased risk included male gender and being older than 30 years, single and unemployed. There may be factors hindering other expatriates from seeking mental health support, such as decreased awareness of mental health problems, stigma and financial issues. It is pertinent to note that Qatar offers NMHH services free of charge. Similarly, treatment for those patients who need formal assessment and in-patient care is also free, and out-patient care is highly subsidised. Perceived implications for employment may be another factor limiting access to the helpline. Studies have highlighted fear among migrants of losing their salary or job if they become ill or take time off work to address health issues and that they are less likely to report mental health problems or seek help for fear of reprisals.^29,30^ Another limiting factor may be the perceived language barrier, as the menu of the NMHH helpline number (16000) is in Arabic and English despite staff being able to speak multiple languages. There is a need for redoubling efforts to improve awareness of mental health problems among the migrant population and employers and to encourage all expatriate populations to seek mental health support and tackle stigma.

### Stigma

Qatar demographics indicate that 64.9% of the country's population is male, but they represented only 42.7% of the study population. This may reflect gender differences in suicidal and self-harm behaviour and stigma among male patients about receiving mental health interventions. Studies conducted in Qatar have indicated negative views of patients with mental illnesses.^31,32^ According to the Qatar Mental Health Strategy Awareness and Attitudes Measure, there are still some barriers to help-seeking, including discomfort discussing mental illness with friends and family owing to stigma and the belief that they will not require any treatment.^32^

Stigma is a barrier to accessing mental health services^33^ that can have serious consequences. A multinational European study revealed lower stigma among people living in countries with higher rates of seeking mental health support.^34^ Initiatives like the NMHH are considered to play an important role in raising awareness of mental health and mental illness. Qatar has introduced mass anti-stigma interventions and that, together with improved access to high-quality mental health services and recruitment of staff from diverse backgrounds at all levels, may encourage people from all backgrounds to seek mental health support.^20,35^ Tackling stigma and improving awareness of the problem of suicide are important factors in preventing suicide.^11^

### Access to mental healthcare

More than half (56.1%) of patients in our sample were not previously known to mental health services and the majority (83.2%) of patients were streamlined into local mental health services to receive ongoing care. This reflects positively on the role of the NMHH service in improving access to mental health services and tackling stigma. Another factor that may have encouraged access is the confidential nature of the service, as patients can choose not to have their NMHH contact/care recorded in the national electronic patients’ records of mainstream health services. However, during consultations the majority of patients agreed to ongoing care through mainstream mental health services. NMHH received calls not only directly from patients, which was the majority group, but also from concerned carers and other professionals, the latter initiating consultations with patients: this reflects the helpline's inclusive approach. These initiatives were helpful not only during the COVID-19 pandemic but also towards the achievement of Qatar National Mental Health and Wellbeing strategy goals of reducing stigma and improving access in the community.^20^

### Presenting symptoms and disorders

The most common symptoms in this sample of patients included suicidal thoughts, depressed mood, disturbed sleep and anxiety and the most common psychiatric disorders were depression, generalised anxiety and mixed anxiety and depressive disorders. Recent use of substances was reported by 8% of patients. Depression and substance use have also been strongly associated with suicide^7,9^ and it reflects the importance of targeting interventions focusing on early identification and management of depression and substance use.

Life stressors such as relationship or family problems, the stress associated with limited social support, the COVID-19 pandemic, and work and living away from family (e.g. among expatriates) were reported by a significant number of patients. Stressful life events have been associated with an increased risk of suicide.^3,7,8,36^ This reflects the importance of paying more attention to stressful life events while undertaking risk assessment and management.

In this study, a history of suicide attempts and the presence of psychotic disorder were significantly associated with the higher priority of risk management, which reflects how the above factors potentially increase the risk of harm to self and a need for increased awareness among healthcare professionals to identify such patients.

### Interventions

Almost three-quarters of patients were offered an intervention by psychiatrists. This is significant considering that Qatar has no established dedicated crisis resolution teams. Interventions were offered more often by psychiatrists if patients suffered from depression and by psychologists if they experienced social stressors. The psychiatrist's role included conducting a biopsychosocial assessment, prescribing, psychoeducation, risk management and referral to appropriate services. They also offered advice to other members of the team on the management of complex cases. In the NMHH, they worked collaboratively with the psychologists and often patients received interventions from both psychiatrist and psychologist. Furthermore, the response time to see a psychiatrist or a psychologist was less than 4 h for the majority of patients, which is impressive. Streamlined access to a truly multidisciplinary team including psychiatrists, psychologists, psychiatric nurses and triage practitioners who are part of the wider mental health services is a unique model for the helpline service which has worked successfully. There is a plan to further consolidate the team, and the NMHH will continue to evolve and offer high-quality services to the population of Qatar.

Nearly all patients were offered non-pharmacological interventions, which included psychoeducation, collaborative problem-solving, crisis resolution strategies, reassurance and signposting to appropriate resources. A smaller proportion (38.5%) received pharmacological interventions and the majority of prescriptions were for the antidepressants escitalopram, sertraline, mirtazapine and fluoxetine, followed by the antipsychotics quetiapine and olanzapine. This corresponds well with the major diagnosis being depression. Antihistamines, melatonin and amitriptyline were recommended for sleep disturbances.

The majority of patients were managed in the community and admission to a psychiatric hospital was advised for only 12% of all the patients. Furthermore, there has been no reported or known incident of completed suicide among this cohort of patients. These findings suggest that the NMHH potentially offers an alternative model of care delivery and more choices to patients to enable them to receive high-quality care in the community. The NMHH team has evolved since its inception into triage, NMHH-integrated interventions, virtual women's mental health, virtual interim psychology, virtual child and adolescent mental health and virtual medications refill services, along with its role in raising awareness to tackle stigma.

### Limitations

This was a retrospective study and therefore dependent on the quality of documentation made by clinicians. The study focuses on the period patients spent under NMHH care, and future studies could include longer-term follow-ups of suicidal patients to investigate outcomes. Some patients, especially if not managed by psychiatrists, did not get a definite diagnosis. Additionally, the generalisability of these results to other countries will depend on their healthcare systems.

### Implications

The results of our study will assist global policy makers in formulating healthcare strategies, resource allocation and designing interventions to prevent suicide and offer early intervention for people who experience suicidal thoughts. Telemedicine, in the form of a service such as NMHH, offers a valuable additional choice to patients and assists in the prevention and management of suicidal behaviour and other mental health difficulties. This study offers valuable insights and paves way for further prospective research incorporating long-term follow-up of patients who received interventions from the helpline services.

## Data Availability

The data are not publicly available owing to ethical restrictions.
